# Retraction: Adaptive Neuro-Fuzzy Determination of the Effect of Experimental Parameters on Vehicle Agent Speed Relative to Vehicle Intruder

**DOI:** 10.1371/journal.pone.0215800

**Published:** 2019-04-23

**Authors:** The PLOS ONE Editors

Following publication, concerns have been raised regarding overlap of text between this article [1] and other previously published works.

There is overlap in the text between the Methodology, Results and Discussion, and Conclusion sections of this article and a previous publication by one of the same authors which presented a different application of the same methodology [2]; that earlier work is not cited and discussed in the *PLOS ONE* article.

Additionally, there is some text overlap in the Introduction and Methodology sections with previously published works by other author groups, including [3,4]. These works are cited, but it is not made clear that text has been re-used verbatim from these sources.

In view of the extent of the overlapping text, the *PLOS ONE* Editors retract this article.

LB-M, IB, SK did not agree with retraction. AWBAW did not respond. SS did not comment on the retraction decision.
